# Are Fish Consumption Advisories for the Great Lakes Adequately Protective against Chemical Mixtures?

**DOI:** 10.1289/EHP104

**Published:** 2016-10-04

**Authors:** Nilima Gandhi, Ken G. Drouillard, George B. Arhonditsis, Sarah B. Gewurtz, Satyendra P. Bhavsar

**Affiliations:** 1 Department of Physical & Environmental Sciences, University of Toronto, Toronto, Ontario, Canada; 2 Great Lakes Institute for Environmental Research, University of Windsor, Windsor, Ontario, Canada; 3 Environmental Monitoring and Reporting Branch, Ontario Ministry of the Environment and Climate Change, Toronto, Ontario, Canada

## Abstract

**Background:**

The North American Great Lakes are home to > 140 types of fish and are famous for recreational and commercial fishing. However, the presence of toxic substances has resulted in the issuance of fish consumption advisories that are typically based on the most restrictive contaminant.

**Objectives:**

We investigated whether these advisories, which typically neglect the existence of a mixture of chemicals and their possible additive adverse effects, are adequately protective of the health of humans consuming fish from the Canadian waters of the Great Lakes.

**Methods:**

Using recent fish contaminant monitoring data collected by the government of Ontario, Canada, we simulated advisories using most-restrictive-contaminant (one-chem) and multi-contaminant additive effect (multi-chem) approaches. The advisories from the two simulations were compared to determine if there is any deficiency in the currently issued advisories.

**Results:**

Approximately half of the advisories currently issued are potentially not adequately protective. Of the four Great Lakes studied, the highest percentage of advisories affected are in Lake Ontario if an additive effect is considered. Many fish that are popular for consumption, such as walleye, salmon, bass and trout, would have noticeably more stringent advisories.

**Conclusions:**

Improvements in the advisories may be needed to ensure that the health of humans consuming fish from the Great Lakes is protected. In this region, total polychlorinated biphenyls (PCBs) and mercury are the major contaminants causing restrictions on consuming fish, whereas dioxins/furans, toxaphene, and mirex/photomirex are of minor concern. Regular monitoring of most organochlorine pesticides and metals in fish can be discontinued.

**Citation:**

Gandhi N, Drouillard KG, Arhonditsis GB, Gewurtz SB, Bhavsar SP. 2017. Are fish consumption advisories for the Great Lakes adequately protective against chemical mixtures? Environ Health Perspect 125:586–593; http://dx.doi.org/10.1289/EHP104

## Introduction

The Great Lakes of North America contain 21% of the world’s surface fresh water and are rich in flora and fauna. The Great Lakes house > 140 types of fish, ranging from panfish to large top-predator fish (Cudmore-Vokey and Crossman 2000). These lakes have supported one of the world’s largest freshwater fisheries for over a century, and their annual contribution is valued at > 5 billion USD [National Oceanic and Atmospheric Administration (NOAA) 2015]. The value and total economic impact of the recreational fisheries far exceed those of the commercial fisheries (NOAA 2015). More than 4 million adults in the U.S. Great Lakes region consume a variety of fish harvested from the Great Lakes every year, and the adults’ consumption is related to their children’s consumption (Turyk et al. 2012). There are > 1 million anglers in Ontario, Canada, and Lakes Erie, Huron, and Ontario are in the top 10 preferred fishing locations for Ontario anglers (Awad 2006; Fisheries and Oceans Canada 2012). Many of the > 160 Aboriginal communities located around the Great Lakes rely on a variety of Great Lakes fish for food (Turyk et al. 2012). For example, a survey of the eating patterns of First Nations people in the Great Lakes basin found that ~84% of the participants consumed on average approximately 20 to 35 fish meals in 1 year [Effects on Aboriginals from the Great Lakes Environment (EAGLE) 2001].

Industrial and agricultural activities have had an impact on the water quality of the Great Lakes through the introduction of toxic substances such as polychlorinated biphenyls (PCBs), mercury, dioxins and furans, and pesticides (Bhavsar et al. 2007c, 2008a, 2010; Murphy et al. 2012). The elevated levels of contaminants in fish have resulted in fish consumption advisories to limit human exposure to contaminants to a safe level [Ontario Ministry of the Environment and Climate Change (OMOECC) 2015; U.S. Environmental Protection Agency (EPA) 2015]. These advisories issued by the province of Ontario for the Canadian waters and by Great Lakes states for the U.S. waters of the Great Lakes are typically based on the most restrictive contaminant [Great Lakes Sport Fish Advisory Task Force (GLSFATF) 1993; OMOECC 2015]. In this approach, fish consumption advisory benchmarks (e.g., see Table S1) are utilized to derive recommended numbers of meals per month for a particular size and type of fish from a specific location, individually for all major contaminants analyzed. The most stringent advisory (i.e., the smallest number of fish meals per month advised) is then selected, and the contaminant causing this restriction is considered the restrictive contaminant. The advisories are presumed to be adequately protective because other contaminants are present but not predominant (GLSFATF 1993), although a consideration of contaminant interactions has been suggested (Bemis and Seegal 1999).

Currently, PCBs are the major drivers of the restrictive fish consumption advisories for the Great Lakes (GLSFATF 1993; OMOECC 2015). Mercury and dioxins/furans are secondary causes of restrictions (Bhavsar et al. 2011; OMOECC 2015). Levels of toxaphene and mirex/photomirex only occasionally cause restrictive advisories (Gandhi et al. 2014, 2015; OMOECC 2015). Exposures to these and to other major contaminants detected in Great Lakes fish can cause a variety of adverse health impacts in humans (Table 1; Murphy et al. 2012). Multiple contaminants can generate health effects that are additive, more than additive (synergistic), or less than additive (i.e., some effects are alleviated). Previous studies have highlighted that PCBs and mercury are the two most important contaminants found in Great Lakes fish (Bhavsar et al. 2007c, 2011; Gandhi et al. 2014, 2015; OMOECC 2015). Although in some cases, the combined effects of PCB and mercury can be less than additive or uncertain, many studies support their additive or synergistic effects (Bemis and Seegal 1999; Fischer et al. 2008; Piedrafita et al. 2008; Powers et al. 2009; Roegge et al. 2004).

**Table 1:** “Do not eat” fish consumption advisory benchmarks used by the province of Ontario, Canada and potential health effects for major contaminants found in Great Lakes fish [OMOECC 2015; Murphy et al. 2012; Agency for Toxic Substances and Disease Registry (ATSDR; http://www.atsdr.cdc.gov/)].

Contaminant	Unit	General population	Sensitive populations	Potential health effects
Mercury
Hg	μg/g	> 1.8	> 0.5	Neurotoxicant; can also damage immune, digestive, and nervous systems
Organic/industrial contaminants
Polychlorinated biphenyls (PCBs)	ng/g	> 844	> 211	Neurotoxicant; affects reproductive and immune systems; developmental effects; potential carcinogen
Dioxin/furan/dioxin-like PCB Toxic Equivalent (TEQ)	pg/g	> 21.6	> 5.4	Neurotoxicant; affects reproductive, immune, and endocrine systems
Perfluorooctane sulfonate (PFOS)	ng/g	> 640	> 160	Potential carcinogen; endocrine disruptions, oxidative stress
Mirex	ng/g	> 657	> 164	Can affect stomach, intestines, liver, kidneys, eyes, thyroid, nervous system, reproductive system
Photomirex	ng/g	> 122	> 31
Toxaphene	ng/g	> 1,877	> 469	Potential carcinogen; convulsions, liver and kidney damage
Total chlordane	ng/g	> 469	> 117	Affects nervous and digestive systems and liver
Total dichlorodiphenyltrichloroethane (DDT)	ng/g	> 5,000	> 5,000	Affects nervous system; potential carcinogen; developmental, reproductive effects
Brominated diphenyl ether 47 (BDE-47)	ng/g	> 939	> 235	Can affect thyroid and liver; behavioral changes; may affect immune system; possible carcinogen; BDE 47 and 99 more toxic than BDE 209
Brominated diphenyl ether 99 (BDE-99)	ng/g	> 939	> 235
Brominated diphenyl ether 153 (BDE-153)	ng/g	> 1,877	> 469
Brominated diphenyl ether 209 (BDE-209)	ng/g	> 65,701	> 16,425
Aldrin + dieldrin	ng/g	> 939	> 235	Potential carcinogen; convulsions, nervous system effects, kidney damage
Hexachlorobenzene (HCB)	ng/g	> 2,534	> 634	Affects nervous system, liver, thyroid; possible carcinogen; endrocine disruptor
Octachlorostyrene (OCS)	ng/g	> 2,910	> 727	Inadequate information available
Metals
Aluminum (Al)	μg/g	> 1,400	> 350	Possible enzyme inhibition; damage to nervous system, Alzheimer disease
Arsenic (As)	μg/g	> 8	> 2	Carcinogen; damage to blood cells and vessels, heart and skin problems
Cadmium (Cd)	μg/g	> 2.8	> 0.7	Probable carcinogen; possible kidney disease, lung damage, and fragile bones
Chromium (Cr)	μg/g	> 14	> 3.5	Chromium (VI) compounds are known human carcinogens; damage to liver, kidney, circulatory and nervous systems, skin irritation
Copper (Cu)	μg/g	> 600	> 150	Essential micronutrient; excess exposure may lead to hemolysis, headache, febrile reactions, prostration, GI symptoms
Lead (Pb)	μg/g	> 16	> 4	Mental retardation, birth defects, psychosis, autism, allergies, dyslexia, weight loss, hyperactivity, paralysis, muscular weakness, brain damage, kidney damage, may even cause death
Manganese (Mn)	μg/g	> 640	> 160	Glucose intolerance, blood clotting, skin problems, skeleton disorders, birth defects, neurological symptoms
Nickel (Ni)	μg/g	> 120	> 30	Damage to lungs, respiratory failure, birth defects, heart disorders, skin problems
Silver (Ag)	μg/g	> 24	> 6	Cardiac abnormalities, permanent damage to brain and nervous system
Selenium (Se)	μg/g	> 24	> 6	Skin and vision problems, shortness of breath, conjunctivitis, vomiting, abdominal pain, diarrhea, enlarged liver
Tin (Sn)	μg/g	> 1.2	> 0.3	Depression, liver damage, immune system problems, chromosomal damage, shortage of red blood cells, brain damage
Zinc (Zn)	μg/g	> 1,400	> 350	Adverse human health effects are rare

It has been recognized that risk assessments of chemical mixtures typically involve substantial uncertainties (U.S. EPA 1986, 2000). When sufficient data on the effects of a chemical mixture are not available, considering additive toxicity is recommended, assuming that the chemicals in the mixture produce adverse effects using the same mode of action (U.S. EPA 1986, 2000). Although PCBs can yield widely varying effects, such as impacts on the reproductive system and development, and are considered carcinogenic, both PCBs and mercury have been recognized as neurotoxicants and can also affect the immune system (Bemis and Seegal 1999; Murphy et al. 2012; Powers et al. 2009). Further, a variety of contaminants found in Great Lakes fish can have many overlapping health effects (Table 1). As such, the assumption of additive toxicity, rather than synergistic or less than additive toxicity, presents a reasonable scenario that also incorporates scientific uncertainty. Our previous work has shown that in the absence of PCBs, mercury, toxaphene, and mirex/photomirex would cause more stringent advisories than they do at present (Bhavsar et al. 2011; Gandhi et al. 2014, 2015). However, it is not clear if consideration of additive effects of the major contaminants known to exist in Great Lake fish would result in only slightly or substantially more stringent advisories.

In this study, we investigated whether the current advisories for the Canadian waters of the Great Lakes are adequately protective of human health when possible additive effects of multiple contaminants are considered. The study also investigated variations in the adequacy of the advisories under this scenario by region, by fish species, and by fish size. Finally, we examined the contributions of individual contaminants to added toxicity from multiple contaminants. Using the recent fish contaminant monitoring data collected by the government of Ontario, Canada, we simulated advisories using both most-restrictive-contaminant and multiple-contaminant approaches. The currently employed most-restrictive-contaminant approach was evaluated by comparing the simulated advisories generated by the two approaches. The outcome of this assessment can inform whether changes need to be made to the current method of issuing fish advisories in order to ensure that the health of humans consuming fish from the Great Lakes is adequately protected.

## Methods

### Data Set

Four of the five lakes in the North American Great Lakes system are shared by the United States and Canada (see Figure S1). The U.S. waters of the Great Lakes are shared by eight states, and nearly all of the Canadian waters of the Great Lakes are within the boundary of the province of Ontario. The OMOECC, in partnership with the Ontario Ministry of Natural Resources and Forestry and other agencies, has monitored contaminants in fish from all parts of the Canadian waters of the Great Lakes since 1970. The measurements collected by the province of Ontario are analytically consistent, and the advisories are based on one method and one set of the benchmarks. As such, we focused our study on the Canadian waters of the Great Lakes.

Because fish contaminant monitoring for different areas and types of fish is conducted on a periodic basis, we considered the measurements collected between 2000 and 2015 as a reasonable recent time period to avoid historical measurements that could have been high for certain legacy contaminants (Bhavsar et al. 2007c, 2008a, 2010) and to maximize data coverage of fish species, their size ranges, and their collection areas. In total, ~145,000 data points for 26 major types of contaminants in skinless, boneless fillets (which can be considered the most edible portion for humans) from 41 types of fish with length (tip of nose to tip of tail) > 15 cm were available. This data set did not include, for example, lipid measurements that are not a part of the advisory calculations. Next, ~85,000 measurements that were below the detection limits (mostly for organochlorine pesticides) were removed because non-detects can have a significant impact on the analysis, which will be elaborated upon in the “Discussion” section. The remaining ~60,000 measurements were then spatially classified into 60 regions used by the OMOECC for the purpose of issuing advisories (see Figure S2; Gandhi et al. 2014) and were used for the advisory simulations.

### Advisory Simulations

The data collected at different time points for each fish species and location were pooled together to create a recent scenario. A power series regression (C = *a* L*^b^*) of fish length (L) versus contaminant concentration (C) was conducted for each available combination of contaminant, fish species, and advisory region, as illustrated in Figure S3. A total of 2,457 regressions were utilized to calculate concentrations of contaminants at 5-cm fish size intervals for each of 575 combinations of species/regions (an average of approximately 5–6 size-based advisories for each of ~10 species per region). These concentrations, standardized to fish lengths (e.g., see Figure S3), were then used to simulate fish consumption advisories.

The methods of advisory simulations using both the current most-restrictive-contaminant (one-chem) approach and the multi-contaminant (multi-chem) approach are illustrated in Table 2 and in “Illustration of advisory calculations using the two approaches: Advisory based on the most-restrictive contaminant approach” and “Advisory based on the multi-contaminant approach” in the Supplemental Material. For the one-chem approach, contaminant concentrations standardized to fish lengths were classified into the advisory categories of 32, 16, 12, 8, 4, 2, 1 or 0 meals/month according to the benchmarks for the general population (GP) and for sensitive populations (SP; women of childbearing age and children) shown in Table S1. Then, the smallest number of meals/month advised for each 5-cm size category for each species and region was selected. For the multi-chem approach, an additive effect was considered. The concentrations standardized to fish lengths were first divided by the contaminant- and population-specific benchmarks for the least stringent (32 meals/month) advisory shown in Table S1. This ratio can be viewed as a contaminant-specific hazard quotient (HQ) for an unrestricted (≥ 32 meals/month) advisory. The HQs for all contaminants for a particular size/species/region/population were summed to calculate a hazard index (HI) reflecting an additive effect of all the contaminants considered. An HI value < 1 would result in an advisory of 32 meals/month. An HI value > 1 would result in an advisory of 0, 1, 2, 4, 8, 12, or 16 meals/month as indicated in “Illustration of advisory calculations using the two approaches: Advisory based on the most-restrictive contaminant approach” and “Advisory based on the multi-contaminant approach” in the Supplemental Material. Because the province of Ontario does not recommend that SP eat fish from the 4- and 2-meals/month advisory categories, those advisories were converted to 0 meals/month (i.e., “do not eat”).

**Table 2:** Illustration of advisory calculations using the one-chem and multi-chem approaches. Detailed explanation is provided in the Supplemental Material.

Approach	Contaminant				
	PCB (ng/g)	Hg (μg/g)	Total TEQ (pg/g)	Toxaphene (ng/g)	Photomirex (ng/g)
Concentration (length standardized)	75	0.81	1.2	75	5
One-chem approach
Individual advisory (meals/month, using benchmarks in Table S1)	8	4	16	16	16
Advisory (meals/month)			4
Multi-chem approach
Benchmark for least restrictive advisory (32 meals/month)	26	0.15	0.7	59	4
HQ (concentration/benchmark for least restrictive advisory)	2.88	5.4	1.71	1.27	1.25
HI (∑HQ)			12.52
32/HI			2.56
Advisory (meals/month)			2
Notes: HI, hazard index; Hg, mercury; HQ, hazard quotient; PCB, polychlorinated biphenyl; TEQ, Toxic Equivalent.					

### Evaluation of the Advisory Approaches

The adequacy of the current approach of issuing advisories based on the most restrictive contaminant (one-chem) was evaluated by comparing advisories generated using the two approaches and classifying the multi-chem advisories into the same or more stringent categories illustrated in Figure S4. The percent contribution of each contaminant to an HI was calculated by dividing contaminant-specific HQs by the HI and multiplying by 100.

## Results

### Impact on the Advisories

**Figure 1:**
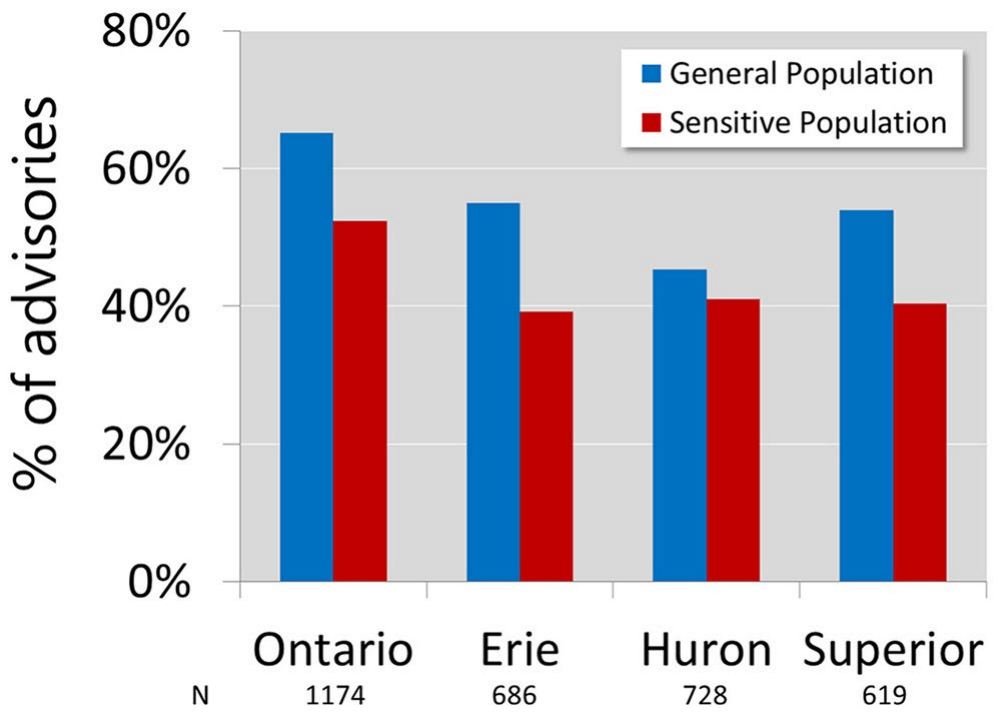
Percentage of the multi-chem approach–based advisories that were more stringent than the one-chem approach. *N* represents the total number of advisories for each population.

Overall, 39–65% of the advisories based on the most restrictive contaminant would be more stringent if the additive adverse effect of major contaminants known to be present in Great Lakes fish were considered (Figure 1). More advisories would be more stringent for GP (45–65%) than for SP (39–52%) (Figure 1).

A breakdown of the advisories indicated that under the multi-chem approach, ≥ 8 meals/month advisories would decline from 58% to 43% of the advisories for GP and from 45% to 28% for SP (Table 3; see also Table S2 and Figure S5). The “do not eat” (i.e., 0 meals/month) advisories would increase from 5% to 10% for GP and from 30% to 47% for SP (Table 3; see also Table S2 and Figure S5). Although the majority of the advisories (43% point of 54% for GP, 32% point of 42% for SP) would be only one category more stringent, which would typically reduce advised meals/month by half, some (10–11%) advisories would be ≥ 2 categories more stringent, suggesting that only one quarter or less of the one-chem–based advised meals/month should be consumed (Table 3; see also Table S3).

**Table 3:** Distribution (in percent) of the advisories (meals/month) simulated using the one-chem and multi-chem approaches.

Population	Multi-chem ↓	One-chem								
		0	1	2	4	8	12	16	32	Total
General population	0	**100%**	59%	4%						10%
	1		**41%**	54%	3%					10%
	2			**42%**	56%	7%	2%			16%
	4				**42%**	71%	31%	2%		21%
	8					**23%**	49%	28%		14%
	12						**19%**	28%	1%	8%
	16							**42%**	12%	10%
	32								**87%**	11%
	Total	5%	7%	13%	16%	15%	10%	21%	13%	100%
Sensitive population	0	**100%**			59%	11%	2%			47%
	4				**41%**	63%	38%	5%		25%
	8					**26%**	33%	33%		12%
	12						**27%**	19%		5%
	16							**43%**	13%	6%
	32								**87%**	5%
	Total	30%			25%	17%	10%	13%	5%	100%
The same advisories from both approaches are presented in bold, and more stringent advisories from the multi-chem approach are highlighted with blue shading. The distributions in the number of advisories are presented in Table S3.										

On a lake-wide basis, adoption of the multi-chem approach would have the least impact on the Lake Huron advisories (41–45% of the advisories would be more stringent), and Lake Erie and Lake Superior would have similar impacts (39–55% of the advisories would be more stringent) (Figure 1). For Lake Ontario, the highest percentage (52–65%) of the advisories would be more stringent (Figure 1). In all regions of the Canadian waters of the Great Lakes, > 20% of the GP advisories would be more stringent except for Lake Erie Wheatley Harbour (LE2b; 0%) and the middle corridor of the St. Lawrence River (LO13; 12%) (see Figure S6). For many (21, or 35% of 60) regions, > 60% of the GP advisories would be more stringent (see Figure S6). Only 8 of 60 (13%) regions would have > 60% of the SP advisories be more stringent, and more regions (9 of 60) would see smaller impacts (< 20% of the advisories would be more stringent) (see Figure S6).

### Contributions of Contaminants

**Figure 2:**
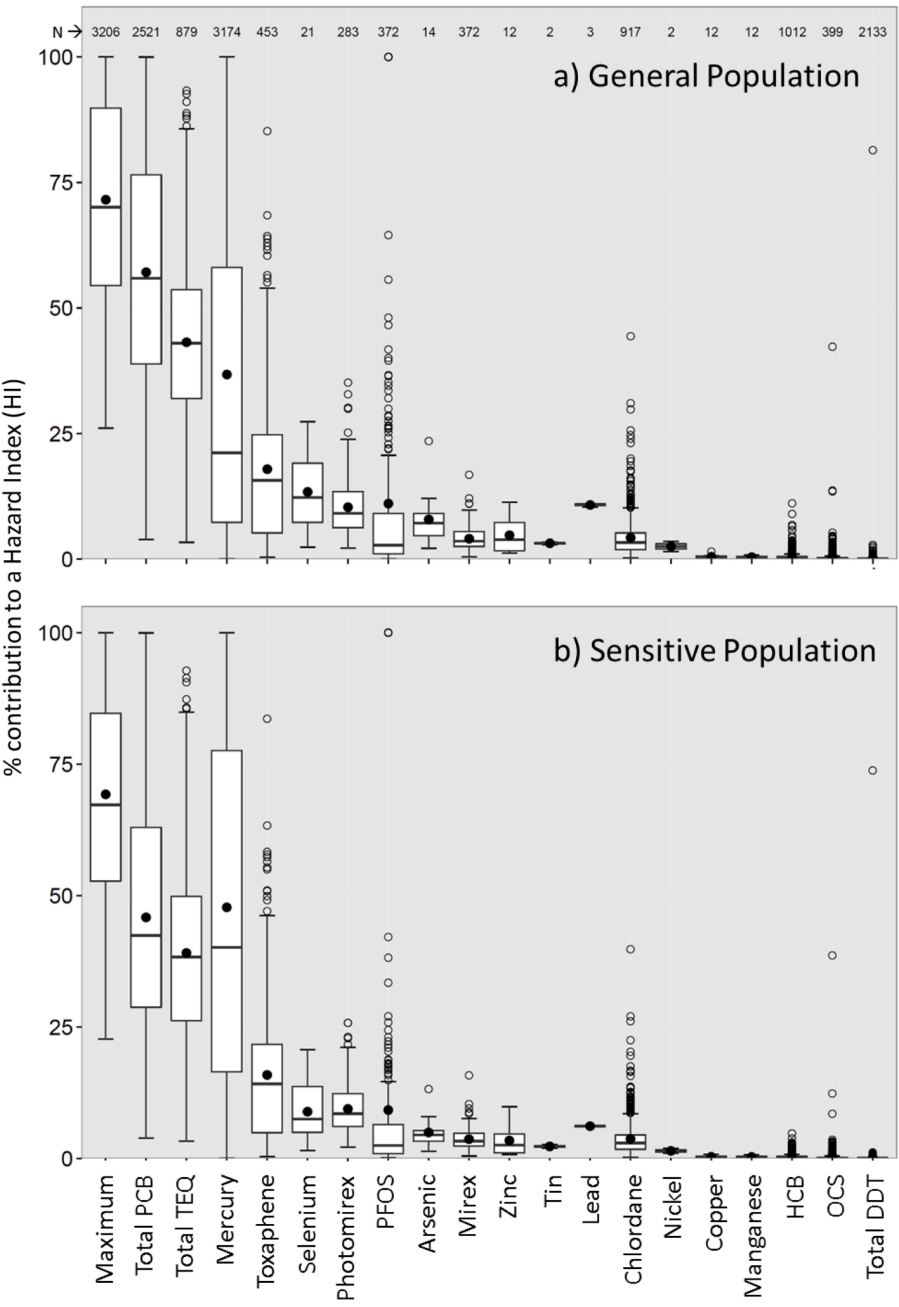
Percent contribution of contaminant-specific hazard quotient (HQ) to the hazard index (HI) calculated using the multi-chem advisory approach for (*A*) the general population and (*B*) sensitive population. The maximum is the highest contribution of an HQ to an HI regardless of contaminant. The solid circle indicates the mean, the line within the box indicates the median, the box indicates the 25th and 75th percentiles, the whiskers indicate the highest and lowest values not classified as statistical outlier values < 1.5 times away from the interquartile range. Nondetect values were excluded. Similar results for a data set that included nondetects are presented in Figure S9. DDT, dichlorodiphenyltrichloroethane; HCB, hexachlorobenzene; OCS, octachlorstyrene; PCB, polychlorinated biphenyl; PFOS, perfluorooctane sulfonate; TEQ, toxic equivalent.

In the multi-chem advisory simulations, a contaminant-specific HQ was calculated, and then HQs for all available contaminants were summed to derive an HI that formulates an advisory. A breakdown of individual HQ contributions to HIs is presented in Figure 2, and the number of multi-chem advisories for which a contaminant was the major contributor to the overall additive effect is presented in Table S4 to provide insight into which contaminants drive the multi-chem–based advisories. The maximum contribution of a contaminant to an HI is, on average, ~70% [standard deviation (SD) 20%]. These results indicate that additive toxicity would be on average ~43% greater [(100 – 70)/70 = 0.43] and could be as high as 300%. Total PCBs would generally be the largest contributor to the additive toxicity (46–57%; SD 21–22%). The average mercury contribution to the advisories for SP would be marginally greater than that of total PCBs at 48% (SD 34%), but lower for the GP at 37% (SD 36%). Toxic equivalent concentrations of dioxins, furans, and dioxin-like PCBs (total TEQ) would on average contribute 43% and 39% (SD 17% for both) for the GP and SP advisories, respectively. Among the dioxins and dioxin-like compounds, most of the contribution to the total TEQ is typically from the dioxin-like PCBs, and dioxin-like PCBs and total PCBs are correlated (Bhavsar et al. 2007a, 2007b, 2008b). As such, PCBs, as a group, would be the major contaminant driving the additive toxicity of the contaminant mixture. Toxaphene would be the only other contaminant with a > 10% average contribution to the additive toxicity for both the GP and for SP. Photomirex and perfluorooctanesulfonic acid (PFOS) would also have some meaningful (average > 10%, *n* > 200) contributions to the additive toxicity for the GP. Some metals would also contribute > 10% on average for the GP advisories; however, these were based on only a few advisories, and nearly all of the HQs for the metals were < 1 (Figure 2; see also Figure S7), implying that their individual levels would allow for fish consumption on a daily basis (i.e., 32 meals/month).

### Fish Species and Size-Specific Differences

**Figure 3:**
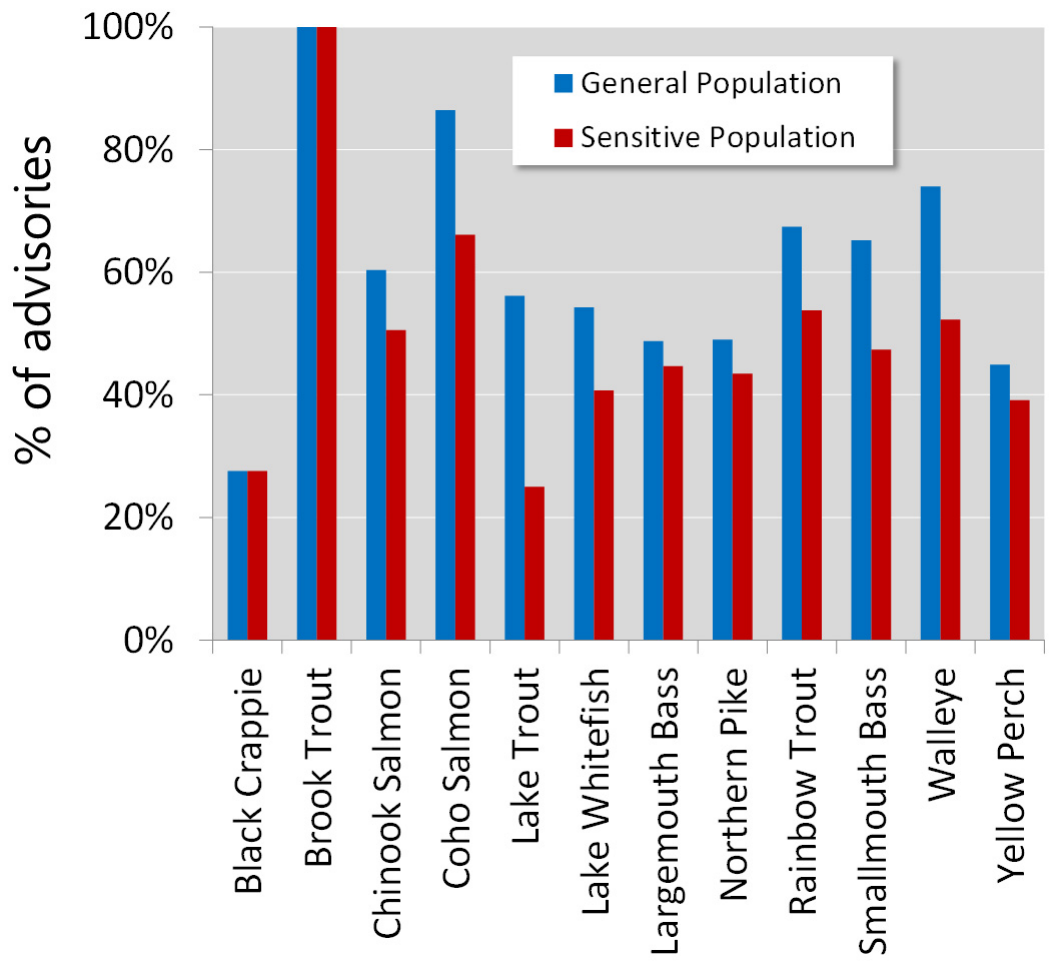
Percentage of the multi-chem approach–based advisories that were more stringent than the one-chem approach for fish favored by anglers in the region. Walleye, lake whitefish, lake trout, perch, and bass are considered the most popular fish among the First Nations communities around the Great Lakes (EAGLE 2001).

Next, we examined if the multi-chem approach affected certain types and sizes of fish differently. Species-specific impacts varied dramatically and generally ranged from 20% to 70% (25th–75th percentile: 33% to 60% for the GP, 26% to 52% for SP; see Table S5). The advisories for walleye, which is the most-favored fish by anglers and Aboriginal peoples in the region (Fisheries and Oceans Canada 2012), would be ~74% and 52% more stringent for the GP and for SP, respectively (Figure 3; see also Table S5). Many other favorite fish such as coho and Chinook salmon, smallmouth bass, and rainbow trout (Awad 2006) would also experience noticeable impacts (> 50% of the advisories woudl be more stringent; Figure 3; see also Table S5). Although some fish size–specific differences were observed for impacts of the multi-chem approach, these differences were relatively moderate (see Table S6). Generally, the largest- and smallest-sized fish were slightly less affected than the medium-sized fish (see Table S6).

### Evaluation of the Method

Finally, we evaluated our method of using data from 2000 to 2015 and grouping the data by advisory regions to create a reasonable recent scenario. Overall, our one-chem simulations indicated 62%, 70%, 54% and 51% of advisories were ≥ 8 meals/month for Lakes Superior, Huron, Erie, and Ontario, respectively (see Figure S8). These results are reasonably similar to the corresponding values of 59%, 58%, 40%, and 42%, respectively, for the real, published advisories (OMOECC 2015). A greater difference for Lake Erie (54% vs. 40%) is likely a result of the exclusion of Lake St. Clair and St. Clair and Detroit River advisories in statistics of the published Lake Erie advisories. Because 62% of the advisories for these excluded areas are ≥ 8 meals/month, inclusion of the St. Clair–Detroit River corridor could have improved the comparison.

**Figure 4:**
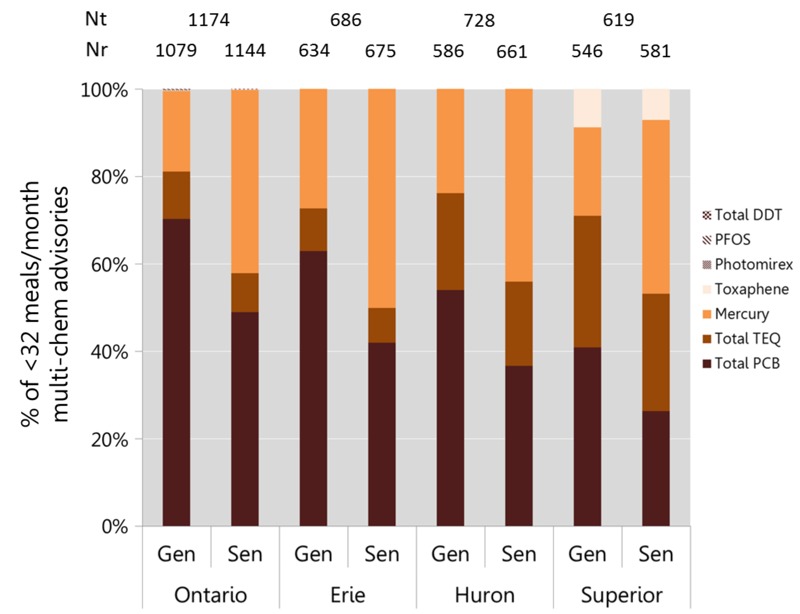
Percent of the multi-chem advisories for which a contaminant is the major contributor to the overall additive effect [assessed as a Hazard Index (HI)]. The contaminants not in the figure were not a major contributor to any HI. Photomirex, perfluorooctane sulfonate (PFOS) and total dichlorodiphenyltrichloroethane (DDT) were major contributors for < 1% of the multi-chem advisories for Lake Ontario only. Gen, general population; Nr, number of advisories that are < 32 meals/month; Nt: total number of advisories; PCB, polychlorinated biphenyl; Sen, sensitive populations; TEQ, Toxic Equivalent.

For all multi-chem–based advisories, contaminants contributing the most to the additive toxicity (i.e., the HI) were tallied. A breakdown of this tally would reflect which contaminants would have caused restrictive advisories under the most-restrictive-contaminant approach of the real published advisories. As shown in Figure 4, approximately 81%, 76%, and 71% of the advisories for Lakes Ontario, Huron, and Superior, respectively, are driven by total PCBs and total TEQ. These results are similar to the values of 88%, 78%, and 68%, respectively, for the published advisories (OMOECC 2015). Mercury drove 18%, 24%, and 20% of the advisories for Lakes Ontario, Huron, and Superior, respectively, in our analysis (Figure 4) and are similar to the corresponding values of 12%, 21%, and 25% for the published advisories (OMOECC 2015). This evaluation indicates that the method used in this study to group data by time and space was reasonably realistic.

Removal of the nondetects was appropriate because their detection limits could be very close to the benchmarks for changing an advisory from 32 to 16 meals/month and could produce HQs close to 1 (e.g., 4 ng/g for both the detection limit and the benchmark of photomirex). Retention of the nondetects in our analysis could have reduced the maximum contribution of a contaminant to an HI from approximately 70% to 50%, on average (Figure 2; see also Figure S9), which translates to a needless average increase of 30% in the additive toxicity.

## Discussion

Approximately half of the Great Lakes advisories based on the most restrictive contaminant would be more stringent if the additive toxicity of the major current contaminants of concern is considered (Figure 1). Although most of these new advisories would result in halving the number of advised meals/month, approximately one tenth of the advisories would recommend comsumption of one quarter or less of the meals/month advised by the one-chem approach (Table 3). Consumption of the more-affected fish as per the one-chem–based advisory would result in four or more times greater exposure to the contaminants than would be considered safe if their additive effects were accounted for. Such unsafe exposures to contaminants may increase the potential for the adverse health effects summarized in Table 1. Therefore, a substantial number of the Great Lakes advisories are likely deficient in protecting the health of human consumers. More advisories are deficient for the GP than for SP likely because a greater number of the current SP advisories are already at 0 meals/month (30% of the SP advisories compared with 5% of the GP advisories) and cannot become more stringent.

Lake Ontario would have the highest percentage of affected advisories, which is in accord with reports of a greater number of contaminants present at elevated levels in Lake Ontario fish than in fish from the other three lakes considered in this study (Murphy et al. 2012). The results indicate that Lake Erie Wheatley Harbour, a former Great Lakes Area of Concern, would not have any impact; however, this finding was a result of only three species monitored in recent times: bigmouth buffalo, common carp and freshwater drum that generally have PCB as a dominating contaminant restricting their consumption. Advisories for Wheatley Harbour yellow perch, which have minor restrictions because of both mercury and PCBs, could show some impact from the multi-chem approach. However, data were not available for yellow perch in Wheatley Harbour during the time period considered.

Our evaluation of the method suggested that the results of this study are reliable; however, availability of more comprehensive monitoring data for a recent period could have minor to moderate influences on the outcome of this analysis. Removal of nondetects avoided needless increases in additive toxicity. Most HQs for total PCBs, dioxins/furans/dioxin-like PCBs, mercury, toxaphene, mirex, and photomirex, and some HQs for chlordane and PFOS were > 1 (see Figure S7). All of the remaining organochlorine pesticides and metals were either below the detection limit or had an HQ < 1, suggesting that their individual levels would not limit consumption of Great Lakes fish beyond a meal a day (i.e., 32 meals/month). Contributions of the HQs of these contaminants to HIs were generally < 10% (except for selenium) and can be considered of no concern. Although only a few above-detection-limit measurements of metals were available in this analysis, we believe that more comprehensive data would not alter the outcome of our analysis because metals typically do not accumulate in fish muscle, and the Great Lakes are not affected by elevated metal levels on a large scale (Jezierska and Witeska 2006; OMOECC 2015). Therefore, the regular monitoring of these other organochlorine pesticides and metals in fish can be discontinued.

This analysis relied on monitoring data collected for the Canadian waters of the Great Lakes. However, it appears that most of the U.S. Great Lakes states typically follow the most-restrictive-contaminant (one-chem) approach. As such, the findings could be, to a great extent, applicable to the U.S. waters of the Great Lakes as well. However, many advisory-issuing agencies incorporate certain conservative steps that could, at least to a certain extent, mitigate the possible deficiencies highlighted in this study. For example, cooking fish on a grill as advised by most agencies could reduce the burden of organic contaminants by 40–60% (e.g., Sherer and Price 1993), but such a reduction is not accounted for in the advisories issued by the government of Ontario for the Canadian waters of the Great Lakes. Furthermore, SP are advised not to eat fish from the 1 and 2 meals/month advisory categories by turning them to 0 meals/month (OMOECC 2015). If we take this into account, 476 of 3,207 (15%) of the SP advisories would not be truly more stringent (see Table S3). The State of Michigan uses the 90th-percentile concentration when a regression between fish length and contaminant concentrations for a sampling event fails to meet a required cut-off for the coefficient of determination (State of Michigan 2014). This method would result in conservative advisories, particularly for smaller-sized fish, because they typically have lower contaminant levels (Gewurtz et al. 2011a, 2011b).

A little conservatism in the advisories is good; however, evolving science stresses the promotion of fish consumption by considering the benefits of consuming fish in addition to the risks (Mozaffarian and Rimm 2006; Neff et al. 2014; Turyk et al. 2012). In addition, possible contamination and lower nutritional quality of other replacement dietary items should be considered. As such, if the current built-in conservatism in calculating one-chem–based advisories mitigates the possible deficiencies highlighted in this study, then more stringent advisories may not be warranted. A better approach could be appropriately accounting for risk (and benefit if possible) at every step of the advisory calculation and removing built-in overconservatism in a scientifically defensible manner.

Lastly, it would be desirable to formulate a generic statistical framework to calculate multi-chemical–based advisories instead of performing the laborious steps utilized in the present study. Namely, rather than developing multiple single-regression models for different contaminants and locations, a more generalizable methodology would involve multivariate regression modeling, a technique that estimates single-regression models with more than one dependent variable to be analyzed simultaneously. In doing so, we will be able to standardize for the fish length while considering the concentrations of all of the contaminants within individual fish. However, both the magnitude and the sign of the covariance among contaminants of concern appear to vary significantly by fish species and location. For example, top predators such as walleye typically have elevated mercury levels, whereas fatty fish from the Great Lakes such as trout and salmon have elevated levels of PCBs (Bhavsar et al. 2011; Sadraddini et al. 2011a, 2011b). Similarly, areas such as the St. Lawrence River generally have higher fish mercury levels, whereas other areas (e.g., Hamilton Harbour) have elevated PCBs (Neff et al. 2013; Visha et al. 2016). Another solution could be to conduct event-specific regressions between length and HQ (instead of length and concentration), but this method would not capture the additive effects of multiple contaminants. Finally, a regression between length and HI could provide a simplified framework; however, such an approach would depend on the availability of measurements for all contaminants in all samples in the event-specific analysis. It may not be possible to obtain all of this information because of the variable costs of analysis (e.g., dioxin analysis is approximately $600–$1,200, whereas mercury analysis is approximately $30–$40 per sample) and it was not possible to do so for the data set used in the present study.

## Conclusion

We investigated whether the current practice of issuing fish consumption advisories for the Great Lakes based on the most-restrictive-contaminant approach is sufficiently protective of the health of humans consuming these fish. Owing to the consistency of the fish contaminant measurements and the area covered, as well as the advisory method and the benchmarks available from OMOECC, we opted to focus our study on the Canadian waters of the Great Lakes. Compared with an individual contaminant, the presence of multiple contaminants can induce a variety of adverse impacts such as less than additive, additive, or synergistic. Assuming additive effects of multiple contaminants, nearly half of the current advisories may not be adequately protective. Many fish such as walleye, salmon, bass, and trout, which are among the favorites for consumption by recreational as well as by subsistence fishers, would have noticeably more stringent advisories under the multi-chem approach. Our findings may also be applicable to the U.S. waters of the Great Lakes. We recommend that agencies issuing advisories evaluate whether any conservative steps presently employed in their advisory methods would protect against the combined effects of multiple contaminants and whether revisions to the issued advisories are necessary.

**Editor’s Note:** In the Advance Publication, the original values for the “Do not eat” advisory benchmarks for dichlorodiphenyltrichloroethane (DDT) were > 93,858 and > 23,465 for the general population and for sensitive populations, respectively. These values were calculated in accordance with the method used by the Province of Ontario using the Tolerable Daily Intake from Health Canada. However, as a protective measure, the Province of Ontario issues a “Do not eat” advisory when DDT is > 5,000. The values displayed in Table 1 have been updated to match those provided by the Province of Ontario.

## Supplemental Material

(1.5 MB) PDF

## References

[r1] Awad E (2006). *The Results of the 2003 Guide to Eating Ontario Sport Fish Questionnaire*..

[r2] Bemis JC, Seegal RF (1999). Polychlorinated biphenyls and methylmercury act synergistically to reduce rat brain dopamine content *in vitro*.. Environ Health Perspect.

[r3] Bhavsar SP, Awad E, Fletcher R, Hayton A, Somers KM, Kolic T (2008a). Temporal trends and spatial distribution of dioxins and furans in lake trout or lake whitefish from the Canadian Great Lakes.. Chemosphere.

[r4] Bhavsar SP, Awad E, Mahon CG, Petro S (2011). Great Lakes fish consumption advisories: is mercury a concern?. Ecotoxicology.

[r5] Bhavsar SP, Fletcher R, Hayton A, Reiner EJ, Jackson DA (2007a). Composition of dioxin-like PCBs in fish: an application for risk assessment.. Environ Sci Technol.

[r6] Bhavsar SP, Gewurtz SB, McGoldrick DJ, Keir MJ, Backus SM (2010). Changes in mercury levels in Great Lakes fish between 1970s and 2007.. Environ Sci Technol.

[r7] Bhavsar SP, Hayton A, Reiner EJ, Jackson DA (2007b). Estimating dioxin-like polychlorinated biphenyl toxic equivalents from total polychlorinated biphenyl measurements in fish.. Environ Toxicol Chem.

[r8] Bhavsar SP, Jackson DA, Hayton A, Reiner EJ, Chen T, Bodnar J (2007c). Are PCB levels in fish from the Canadian Great Lakes still declining?. J Great Lakes Res.

[r9] Bhavsar SP, Reiner EJ, Hayton A, Fletcher R, MacPherson K (2008b). Converting Toxic Equivalents (TEQ) of dioxins and dioxin-like compounds in fish from one Toxic Equivalency Factor (TEF) scheme to another.. Environ Int.

[r10] Cudmore-Vokey B, Crossman EJ (2000). *Checklists of the Fish Fauna of the Laurentian Great Lakes and Their Connecting Channels*. Canadian Manuscript Report of Fisheries and Aquatic Sciences 2550.. https://pdfs.semanticscholar.org/c5bb/d5e7fbedd1232a15a97c34666a5c6d34cf48.pdf.

[r11] EAGLE (Effects on Aboriginals from the Great Lakes Environment) (2001). EAGLE Eating Patterns Survey.. http://www.chiefs-of-ontario.org/sites/default/files/files/epsfin2.pdf.

[r12] Fischer C, Fredriksson A, Eriksson P (2008). Neonatal co-exposure to low doses of an ortho-PCB (PCB 153) and methyl mercury exacerbate defective developmental neurobehavior in mice.. Toxicology.

[r13] Fisheries and Oceans Canada (2012). *2010 Survey of Recreational Fishing in Canada*.. http://www.dfo-mpo.gc.ca/stats/rec/can/2010/RECFISH2010_ENG.pdf.

[r14] Gandhi N, Bhavsar SP, Tang RWK, Drouillard KG, Arhonditsis GB (2014). Significance of toxaphene in Great Lakes fish consumption advisories.. J Great Lakes Res.

[r15] Gandhi N, Tang RWK, Bhavsar SP, Reiner EJ, Morse D, Arhonditsis GB (2015). Is mirex still a contaminant of concern for the North American Great Lakes?. J Great Lakes Res.

[r16] Gewurtz SB, Backus SM, Bhavsar SP, McGoldrick DJ, de Solla SR, Murphy EW (2011a). Contaminant biomonitoring programs in the Great Lakes region: review of approaches and critical factors.. Environ Rev.

[r17] Gewurtz SB, Bhavsar SP, Fletcher R (2011b). Influence of fish size and sex on mercury/PCB concentration: importance for fish consumption advisories.. Environ Int.

[r18] GLSFATF (Great Lakes Sport Fish Advisory Task Force) (1993). *Protocol for a Uniform Great Lakes Sports Fish Consumption Advisory*.. http://www.health.state.mn.us/divs/eh/fish/consortium/pastprojects/pcbprotocol.pdf.

[r19] Jezierska B, Witeska M (2006). The metal uptake and accumulation in fish living in polluted waters. In: *Soil and Water Pollution Monitoring, Protection and Remediation*. Twardowska I, Allen HE, Häggblom MM, Stefaniak S, eds. NATO Science Series. Vol. 69..

[r20] Mozaffarian D, Rimm E (2006). Fish intake, contaminants, and human health: evaluating the risks and the benefits.. JAMA.

[r21] Murphy CA, Bhavsar SP, Gandhi N (2012). Contaminants in Great Lakes fish: historic, current, and emerging concerns. In: *Great Lakes Fisheries Policy and Management: A Binational Perspective*. 2nd ed. Taylor WW, Lynch AJ, Leonard NJ, eds..

[r22] Neff MR, Bhavsar SP, Ni FJ, Carpenter DO, Drouillard K, Fisk AT (2014). Risk-benefit of consuming Lake Erie fish.. Environ Res.

[r23] Neff MR, Robinson JM, Bhavsar SP (2013). Assessment of fish mercury levels in the upper St. Lawrence River, Canada.. J Great Lakes Res.

[r24] NOAA (National Oceanic and Atmospheric Administration) (2015). About Our Lakes: Economy.. https://www.glerl.noaa.gov/education/ourlakes/economy.html.

[r25] OMOECC (Ontario Ministry of the Environment and Climate Change) (2015). *2015–2016 Guide to Eating Ontario Fish*; *Guide de consommation du poisson de l’Ontario*.. https://dr6j45jk9xcmk.cloudfront.net/documents/4460/fishguide2015-final-aoda-en-final.pdf.

[r26] Piedrafita B, Erceg S, Cauli O, Felipo V (2008). Developmental exposure to polychlorinated biphenyls or methylmercury, but not to its combination, impairs the glutamate–nitric oxide–cyclic GMP pathway and learning in 3-month-old rats.. Neuroscience.

[r27] Powers BE Poon E Sable HJK Schantz SL 2009 Developmental exposure to PCBs, MeHG, or both: long-term effects on auditory function. Environ Health Perspect 117 1101 1107, doi:10.1289/ehp.0800428 19654920 PMC2717137

[r28] Roegge CS, Wang VC, Powers BE, Klintsova AY, Villareal S, Greenough WT (2004). Motor impairment in rats exposed to PCBs and methylmercury during early development.. Toxicol Sci.

[r29] Sadraddini S Azim ME Shimoda Y Bhavsar SP Drouillard KG Backus SM 2011a A Bayesian assessment of the PCB temporal trends in Lake Erie fish communities. J Great Lakes Res 37 507 520, doi:10.1016/j.jglr.2011.06.005

[r30] Sadraddini S, Azim ME, Shimoda Y, Mahmood M, Bhavsar SP, Backus SM (2011b). Temporal PCB and mercury trends in Lake Erie fish communities: a dynamic linear modeling analysis.. Ecotoxicol Environ Saf.

[r31] Sherer RA, Price PS (1993). The effect of cooking processes on PCB levels in edible fish tissue.. Qual Assur.

[r32] State of Michigan (2014). *Michigan Fish Consumption Advisory Program: Guidance Document*.. http://www.michigan.gov/documents/mdch/MDCH_MFCAP_Guidance_Document_417043_7.pdf.

[r33] Turyk ME Bhavsar SP Bowerman W Boysen E Clark M Diamond M 2012 Risks and benefits of consumption of Great Lakes fish. Environ Health Perspect 120 11 18, doi:10.1289/ehp.1003396 21947562 PMC3261933

[r34] U.S. EPA (U.S. Environmental Protection Agency) (1986). *Guidelines for the Health Risk Assessment of Chemical Mixtures*. EPA/630/R-98/002.. https://ofmpub.epa.gov/eims/eimscomm.getfile?p_download_id=4487.

[r35] U.S. EPA (2000). *Supplementary Guidance for Conducting Health Risk Assessment Of Chemical Mixtures*. EPA/630/R-00/002.. https://ofmpub.epa.gov/eims/eimscomm.getfile?p_download_id=4486.

[r36] U.S. EPA (2015). Choose Fish and Shellfish Wisely: Fish and Shellfish Advisories and Safe Eating Guidelines.. https://www.epa.gov/choose-fish-and-shellfish-wisely/fish-and-shellfish-advisories-and-safe-eating-guidelines.

[r37] Visha A, Gandhi N, Bhavsar SP, Arhonditsis GB (2016). Guiding fish consumption advisories for Lake Ontario: a Bayesian hierarchical approach.. J Great Lakes Res.

