# Supplementary material for: Are Fish Consumption Advisories for the Great Lakes Adequately Protective against Chemical Mixtures?
Source: Environ Health Perspect. 2016 Oct 4;125(4):586–93. doi: 10.1289/EHP104 (PMC5381969; doi:10.1289/EHP104)

Environ Health Perspect
DOI: 10.1289/EHP104

Note to readers with disabilities: *EHP* strives to ensure that all journal content is accessible to all readers. However, some figures and Supplemental Material published in *EHP* articles may not conform to 508 standards due to the complexity of the information being presented. If you need assistance accessing journal content, please contact ehponline@niehs.nih.gov. Our staff will work with you to assess and meet your accessibility needs within 3 working days.

Supplemental Material

Are Fish Consumption Advisories for the Great Lakes Adequately Protective from Chemical Mixture?

Nilima Gandhi, Ken G. Drouillard, George B. Arhonditsis, Sarah B. Gewurtz, and Satyendra P. Bhavsar

Table of Contents

Illustration of advisory calculations using the two approaches

Advisory based on the most-restrictive contaminant approach

Advisory based on the multi-contaminant approach

Table S1: Fish consumption advisory benchmarks used by the Province of Ontario, Canada (OMOECC 2015). The sensitive population is advised not to eat fish from the 1 and 2 meals/month categories by turning them to 0 meal/month.

Table S2: Breakdown of the simulated fish consumption advisories for the general and sensitive populations using the one-chem and multi-chem approaches.

Table S3: Distribution (in number) of the advisories (meals/month) simulated using the one-chem and multi-chem approaches. The same advisories from both approaches are presented in bold, while more stringent advisories from the multi-contaminant approach are highlighted with gray shading. The distribution in percentage (%) of advisories is presented in Table 1.

Table S4: Number of the multi-chem advisories for which a contaminant was the major contributor to the overall additive effect (assessed as a Hazard Index – HI).

Table S5: Breakdown (by species) of number and percentage of the multi-chem approach based advisories that were the same or more stringent compared to the one-chem approach. Species

that are considered popular among anglers (Awad 2006) are highlighted in bold. Walleye, Lake Whitefish, Lake Trout, Perch and Bass can be considered most popular among the First Nations communities around the Great Lakes (EAGLE 2001).

Table S6: Breakdown (by fish size) of number and percentage of the multi-chem approach based advisories that were the same or more stringent compared to the one-chem approach.

Figure S1: Map of the North American Great Lakes (map created using R statistical software)

Figure S2: Map of the Canadian waters of the Great Lakes divided into blocks for fish consumption advisory purposes by the OMOECC (OMOECC 2015) (adapted from Gandhi et al. 2014, with permission of Elsevier).

Figure S3: Illustration of standardising contaminant concentrations to fish lengths at 5 cm intervals using a power series regression. Circles are for individual measurements for a particular contaminant in samples of a fish species collected from a block (Figure S2) between 2000 and 2015. This regression resulted in 12 concentrations of the contaminant at 5 cm fish size intervals for the species/blocks.

Figure S4: Illustration of a comparison of advisories from the one-chem and multi-chem approaches. The comparison was block-, species- and population- (general and sensitive) specific. The yellow highlighted multi-chem advisories were classified as “more stringent”, while the remaining multi-chem advisories were classified as the same.

Figure S5: Distribution (in number) of the advisories (meals/month) simulated using the one-chem and multi-chem approaches.

Figure S6: Breakdown (by advisory regions) of percentage of the multi-chem approach based advisories that were more stringent compared to the one-chem approach. Advisory regions are shown in Figure S2. LS: Lake Superior; SMR: St. Mary’s River; NC: North Channel (Lake Huron); GB: Georgian Bay (Lake Huron); LE: Lake Erie; LO: Lake Ontario.

Figure S7: Contaminant-specific Hazard Quotient (HQ) calculated in the multi-chem advisory simulations. The line within the box indicates a median, the box indicates 25th and 75th percentile values, the whiskers indicate the highest and lowest values not classified as statistical outlier values more than 1.5 times away from the interquartile range. Red dotted line indicates an HQ of 1.

Figure S8:

Figure S9: Percent contribution of contaminant-specific Hazard Quotient (HQ) to the Hazard Index (HI) calculated in the multi-chem advisory approach. Maximum is for the highest contribution of an HQ to HI regardless of a contaminant. The solid circle indicates a mean, the line within the box indicates a median, the box indicates 25th and 75th percentile values, the

whiskers indicate the highest and lowest values not classified as statistical outlier values more than 1.5 times away from the interquartile range. Non-detect values were excluded. Similar results for a dataset that included non-detects are presented in Figure 2.

Illustration of advisory calculations using the two approaches

Advisory based on the most-restrictive contaminant approach

Contaminant	PCB	Mercury	Total TEQ	Toxaphene	Photomirex
Unit	ng/g	µg/g	pg/g	ng/g	ng/g
Concentration (length standardized)	75	0.81	1.2	75	5
Individual Advisory (meals/month, based on benchmarks)	8	4	16	16	16
Advisory (meals/month, Most-restrictive contaminant)			4		

As shown in the table above, concentrations of contaminants measured above the detection limits are first standardized to a particular fish length as per illustration in Figure S3. These concentrations are then classified into advisory categories (e.g., Figure S4) as per the benchmarks shown in Table S1. Based on the most restrictive contaminant (in this case, mercury), the least number of meals/month advised is selected as the final advisory (in this case, 4 meals/month).

Advisory based on the multi-contaminant approach

Contaminant	PCB	Mercury	Total TEQ	Toxaphene	Photomirex
Unit	ng/g	µg/g	pg/g	ng/g	ng/g
Concentration (length standardized)	75	0.81	1.2	75	5
Benchmark for least restrictive advisory (32 meals/month)	26	0.15	0.7	59	4
HQ (Concentration/Benchmark for least restrictive advisory)	2.88	5.40	1.71	1.27	1.25
HI (∑HQ)			12.52		
32/HI			2.56		
Advisory (meals/month, Multi-contaminant)			2		

As shown in the table above, concentrations of contaminants measured above the detection limits are first standardized to a particular fish length as per illustration in Figure S3. These concentrations are then divided by the corresponding benchmark for the least stringent advisory (i.e., 32 meals/month; Table S1) to calculate contaminant-specific Hazard Quotients (HQs). The HQs are then summed to derive a Hazard Index (in this case, 12.52). The HI presents the overall additive toxic equivalent concentration of the mixture relative to unity toxicity before a 32 meals/month advisory is changed

to 16 meals/month. As such, next, a calculation of 32/HI presents how many meals/month would be suitable to keep the additive toxicity of the chemical mixture to unity (in this case, 32/12.52 = 2.56). Since the closest lower end of meals/month advisory category (out of 32, 16, 12, 8, 4, 2, 1, 0 meals/month) for 2.56 is 2, the final advisory for the multi-contaminant approach would be 2 meals/month.

The above example table highlights that a 4 meals/month advisory from the most-restrictive contaminant approach could become a 2 meals/month advisory if the multi-contaminant approach would be used.

Table S1: Fish consumption advisory benchmarks used by the Province of Ontario, Canada (OMOECC 2015). The sensitive population is advised not to eat fish from the 1 and 2 meals/month categories by turning them to 0 meal/month.

		Meals per month	0 (do not eat)	1	2	4	8	12	16	32
Mercury	Hg; ug/g	Sensitive Popn	>0.5			0.25-0.5	0.16-0.25	0.12-0.16	0.06-0.12	<0.06
		General Popn	>1.8		1.2-1.8	0.6-1.2	0.4-0.6	0.3-0.4	0.15-0.3	<0.15
Organic / Industrial contaminants	Polychlorinated biphenyl (PCB)	ng/g	>844	422-844	211-422	105-211	70-105	53-70	26-53	<26
	Dioxin/Furan/dlPCB Toxic Equivalent (TEQ)	pg/g	>21.6	10.8-21.6	5.4-10.8	2.7-5.4	1.8-2.7	1.3-1.8	0.7-1.3	<0.7
	Perfluorooctane Sulfonate (PFOS)	ng/g	>640	320-640	160-320	80-160	53-80	40-53	20-40	<20
	Mirex	ng/g	>657	329-657	164-329	82-164	55-82	41-55	21-41	<21
	Photomirex	ng/g	>122	61-122	31-61	15-31	10-15	8-10	4-8	<4
	Toxaphene	ng/g	>1877	939-1877	469-939	235-469	156-235	117-156	59-117	<59
	Total Chlordane	ng/g	>469	235-469	117-235	59-117	39-59	29-39	15-29	<15
	Total ichlorodiphenyltrichloroethane (DDT)	ng/g	>93858	46929-93858	23465-46929	11732-23465	7822-11732	5866-7822	2933-5866	<2933
	Brominated diphenyl ether 47 (BDE-47)	ng/g	>939	469-939	235-469	117-235	78-117	59-78	29-59	<29
	Brominated diphenyl ether 99 (BDE-99)	ng/g	>939	469-939	235-469	117-235	78-117	59-78	29-59	<29
	Brominated diphenyl ether 153 (BDE-153)	ng/g	>1877	939-1877	469-939	235-469	156-235	117-156	59-117	<59
	Brominated diphenyl ether 209 (BDE-209)	ng/g	>65701	32850-65701	16425-32850	8213-16425	5475-8213	4106-5475	2053-4106	<2053
	Aldrin+Dieldrin	ng/g	>939	469-939	235-469	117-235	78-117	59-78	29-59	<29
	Hexachlorobenzene (HCB)	ng/g	>2534	1267-2534	634-1267	317-634	211-317	158-211	79-158	<79
	Octachlorostyrene (OCS)	ng/g	>2910	1455-2910	727-1455	364-727	242-364	182-242	91-182	<91
Metals	Aluminum (Al)	ug/g	>1400	700-1400	350-700	175-350	117-175	88-117	44-88	<44
	Arsenic (As)	ug/g	>8	4-8	2-4	1-2	0.67-1.00	0.50-0.67	0.25-0.50	<0.25
	Cadmium (Cd)	ug/g	>2.8	1.4-2.8	0.7-1.4	0.35-0.70	0.23-0.35	0.18-0.23	0.09-0.18	<0.09
	Chromium (Cr)	ug/g	>14	7-14	3.5-7.0	1.75-3.50	1.17-1.75	0.88-1.17	0.44-0.88	<0.44
	Copper (Cu)	ug/g	>600	300-600	150-300	75-150	50-75	38-50	19-38	<19
	Lead (Pb)	ug/g	>16	8-16	4-8	2-4	1.33-2	1.0-1.33	0.5-1.0	<0.5
	Manganese (Mn)	ug/g	>640	320-640	160-320	80-160	53-80	40-53	20-40	<20
	Nickel (Ni)	ug/g	>120	60-120	30-60	15-30	10-15	7.5-10	3.75-7.5	<3.75
	Silver (Ag)	ug/g	>24	12-24	6-12	3-6	2-3	1.5-2	0.75-1.5	<0.75
	Selenium (Se)	ug/g	>24	12-24	6-12	3-6	2-3	1.5-2	0.75-1.5	<0.75
	Tin (Sn)	ug/g	>1.2	0.6-1.2	0.3-0.6	0.15-0.3	0.10-0.15	0.075-0.10	0.038-0.075	<0.038
	Zinc (Zn)	ug/g	>1400	700-1400	350-700	175-350	117-175	88-117	44-88	<44

Table S2: Breakdown of the simulated fish consumption advisories for the general and sensitive populations using the one-chem and multi-chem approaches.

**a)**	**General**		**Sensitive**	
**Meal/month**	**One-chem**	**Multi-chem**	**One-chem**	**Multi-chem**
0	167	324	966	1505
1	237	335		
2	412	499		
4	524	660	807	814
8	468	448	537	380
12	318	251	320	164
16	667	328	410	198
32	414	362	167	146
Total	3207	3207	3207	3207

**b)**	**General**		**Sensitive**	
**Meal/month**	**One-chem**	**Multi-chem**	**One-chem**	**Multi-chem**
0	5%	10%	30%	47%
1	7%	10%		
2	13%	16%		
4	16%	21%	25%	25%
8	15%	14%	17%	12%
12	10%	8%	10%	5%
16	21%	10%	13%	6%
32	13%	11%	5%	5%
Total	100%	100%	100%	100%

Table S3: Distribution (in number) of the advisories (meals/month) simulated using the one-chem and multi-chem approaches. The same advisories from both approaches are presented in bold, while more stringent advisories from the multi-contaminant approach are highlighted with gray shading. The distribution in percentage (%) of advisories is presented in Table 1.

		**Most restrictive-contaminant (one-chem) advi**								
	**Multi-cont Advi ↓**	**0**	**1**	**2**	**4**	**8**	**12**	**16**	**32**	**Total**
**General Popn**	0	**167**	141	16						324
	1		**96**	224	15					335
	2			**172**	291	31	5			499
	4				**218**	331	97	14		660
	8					**106**	155	187		448
	12						**61**	186	4	251
	16							**280**	48	328
	32								**362**	362
	Total	167	237	412	524	468	318	667	414	3207
**Sensitive Popn**	0	**966**			476	57	6			1505
	4				**331**	340	121	22		814
	8					**140**	106	134		380
	12						**87**	77		164
	16							**177**	21	198
	32								**146**	146
	Total	966			807	537	320	410	167	3207

Table S4: Number of the multi-chem advisories for which a contaminant was the major contributor to the overall additive effect (assessed as a Hazard Index – HI).

	Superior	Huron	Erie	Ontario	Total
**General Popn**					
Total PCB	220	308	395	743	1666
Total TEQ	162	126	61	114	463
Mercury	109	136	172	195	612
Toxaphene	47			1	48
Photomirex				2	2
PFOS				1	1
Total DDT				1	1
**Sensitive Popn**					
Total PCB	152	240	282	555	1229
Total TEQ	156	126	53	101	436
Mercury	230	289	337	475	1331
Toxaphene	41			1	42
Photomirex					
PFOS				1	1
Total DDT				1	1

Table S5: Breakdown (by species) of number and percentage of the multi-chem approach based advisories that were the same or more stringent compared to the one-chem approach. Species that are considered popular among anglers (Awad 2006) are highlighted in bold. Walleye, Lake Whitefish, Lake Trout, Perch and Bass can be considered most popular among the First Nations communities around the Great Lakes (EAGLE 2001).

		General Popn		Sensitive Popn		General Popn		Sensitive Popn	
**Species**	**Total**	**More Stringent**	**Same**	**More Stringent**	**Same**	**More Stringent**	**Same**	**More Stringent**	**Same**
American Eel	6		6		6	0%	100%	0%	100%
Atlantic Salmon	10	7	3		10	70%	30%	0%	100%
Bigmouth Buffalo	3		3		3	0%	100%	0%	100%
**Black Crappie**	**29**	**8**	**21**	**8**	**21**	**28%**	**72%**	**28%**	**72%**
Bloater	9	3	6	3	6	33%	67%	33%	67%
Bluegill	18	10	8	8	10	56%	44%	44%	56%
Bowfin	7	4	3	2	5	57%	43%	29%	71%
**Brook Trout**	**5**	**5**		**5**		**100%**	**0%**	**100%**	**0%**
Brown Bullhead	97	56	41	51	46	58%	42%	53%	47%
Brown Trout	79	51	28	12	67	65%	35%	15%	85%
Channel Catfish	115	52	63	28	87	45%	55%	24%	76%
**Chinook Salmon**	**174**	**105**	**69**	**88**	**86**	**60%**	**40%**	**51%**	**49%**
Chub	9	4	5	6	3	44%	56%	67%	33%
Cisco(Lake Herring)	35	23	12	26	9	66%	34%	74%	26%
**Coho Salmon**	**59**	**51**	**8**	**39**	**20**	**86%**	**14%**	**66%**	**34%**
Common Carp	211	100	111	79	132	47%	53%	37%	63%
Freshwater Drum	132	67	65	50	82	51%	49%	38%	62%
Gizzard Shad	10	2	8	6	4	20%	80%	60%	40%
Goldfish	4		4		4	0%	100%	0%	100%
Humper-Banker Lake Trout	17	10	7	5	12	59%	41%	29%	71%

**Lake Trout**	**260**	**146**	**114**	**65**	**195**	**56%**	**44%**	**25%**	**75%**
**Lake Whitefish**	**199**	**108**	**91**	**81**	**118**	**54%**	**46%**	**41%**	**59%**
**Largemouth Bass**	**123**	**60**	**63**	**55**	**68**	**49%**	**51%**	**45%**	**55%**
Ling (Burbot)	53	17	36	14	39	32%	68%	26%	74%
Longnose Sucker	54	37	17	35	19	69%	31%	65%	35%
**Northern Pike**	**253**	**124**	**129**	**110**	**143**	**49%**	**51%**	**43%**	**57%**
Pink Salmon	27	16	11	19	8	59%	41%	70%	30%
Pumpkinseed	14	3	11	3	11	21%	79%	21%	79%
Rainbow Smelt	1		1	1		0%	100%	100%	0%
**Rainbow Trout**	**184**	**124**	**60**	**99**	**85**	**67%**	**33%**	**54%**	**46%**
Redhorse Sucker	37	23	14	18	19	62%	38%	49%	51%
Rock Bass	62	32	30	27	35	52%	48%	44%	56%
Round Whitefish	46	13	33	19	27	28%	72%	41%	59%
Salmon Hybrid	3		3		3	0%	100%	0%	100%
Siscowet	12	5	7	2	10	42%	58%	17%	83%
**Smallmouth Bass**	**135**	**88**	**47**	**64**	**71**	**65%**	**35%**	**47%**	**53%**
**Walleye**	**304**	**225**	**79**	**159**	**145**	**74%**	**26%**	**52%**	**48%**
White Bass	45	18	27	12	33	40%	60%	27%	73%
White Perch	51	19	32	20	31	37%	63%	39%	61%
White Sucker	177	71	106	87	90	40%	60%	49%	51%
**Yellow Perch**	**138**	**62**	**76**	**54**	**84**	**45%**	**55%**	**39%**	**61%**
Total	3207	1749	1458	1360	1847	55%	45%	42%	58%

Table S6: Breakdown (by fish size) of number and percentage of the multi-chem approach based advisories that were the same or more stringent compared to the one-chem approach.

		General Popn		Sensitive Popn		General Popn		Sensitive Popn	
**Fish Size (cm)**	**Total**	**More Stringent**	**Same**	**More Stringent**	**Same**	**More Stringent**	**Same**	**More Stringent**	**Same**
15-20	175	48	127	71	104	27%	73%	41%	59%
20-25	248	97	151	107	141	39%	61%	43%	57%
25-30	274	112	162	117	157	41%	59%	43%	57%
30-35	304	150	154	147	157	49%	51%	48%	52%
35-40	321	186	135	171	150	58%	42%	53%	47%
40-45	322	200	122	176	146	62%	38%	55%	45%
45-50	317	199	118	164	153	63%	37%	52%	48%
50-55	289	184	105	130	159	64%	36%	45%	55%
55-60	255	170	85	101	154	67%	33%	40%	60%
60-65	227	162	65	70	157	71%	29%	31%	69%
65-70	192	108	84	52	140	56%	44%	27%	73%
70-75	155	88	67	39	116	57%	43%	25%	75%
75+	128	45	83	15	113	35%	65%	12%	88%
**Total**	3207	1749	1458	1360	1847	55%	45%	42%	58%

Figure S1: Map of the North American Great Lakes (map created using R statistical software)

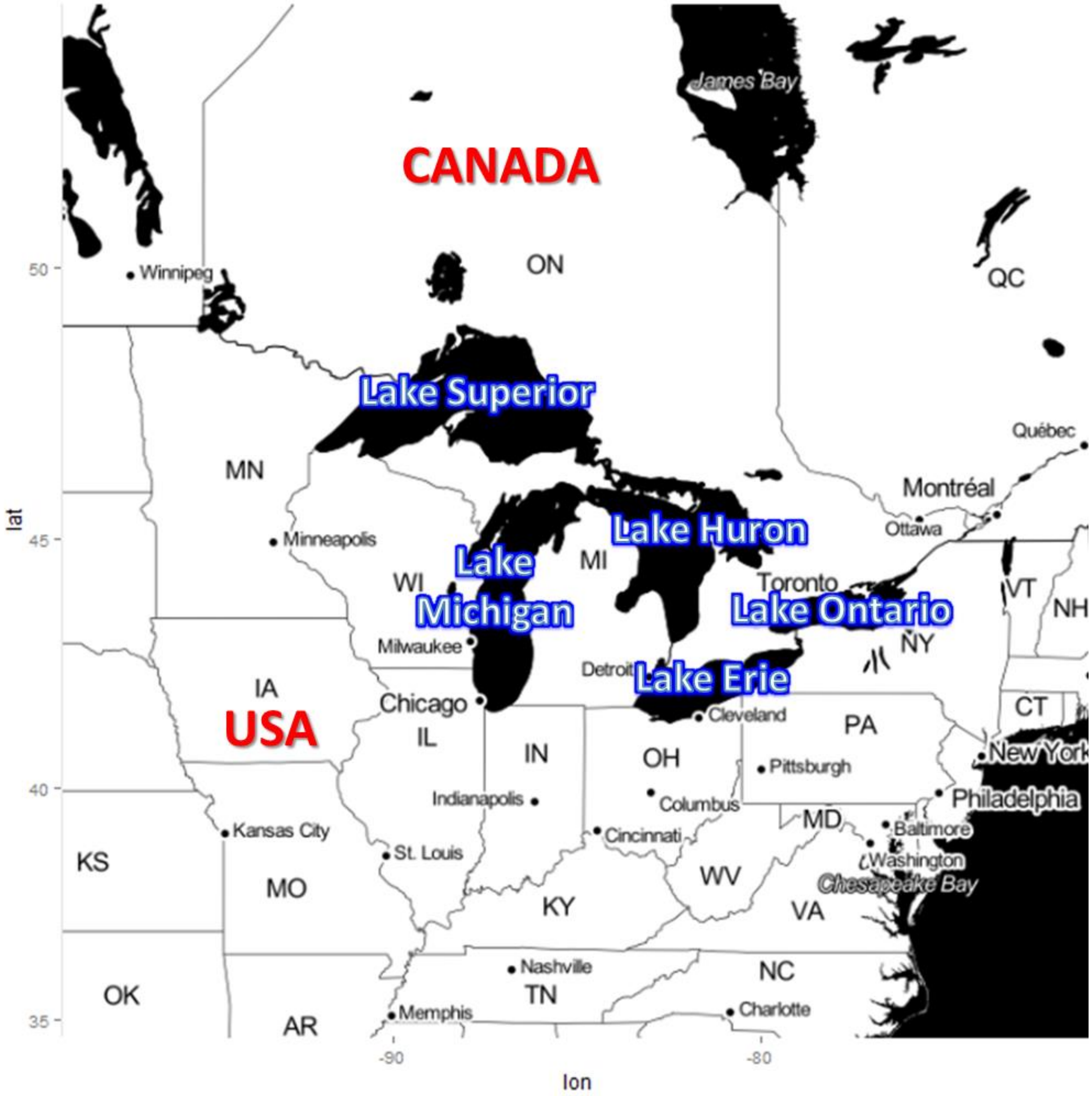

Figure S2: Map of the Canadian waters of the Great Lakes divided into blocks for fish consumption advisory purposes by the OMOECC (OMOECC 2015) (adapted from Gandhi et al. 2014, with permission of Elsevier).

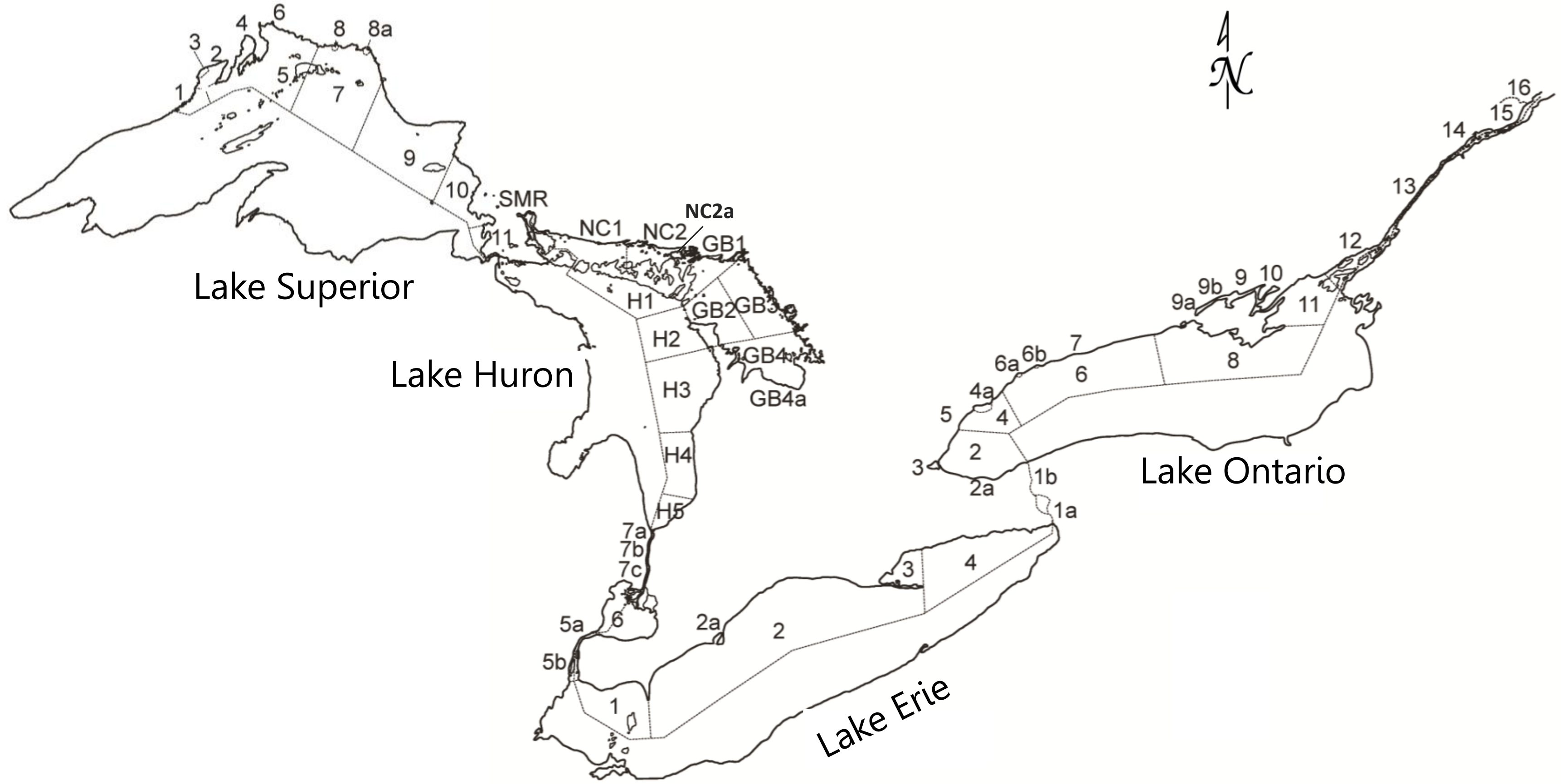

Figure S3: Illustration of standardising contaminant concentrations to fish lengths at 5 cm intervals using a power series regression. Circles are for individual measurements for a particular contaminant in samples of a fish species collected from a block (Figure S2) between 2000 and 2015. This regression resulted in 12 concentrations of the contaminant at 5 cm fish size intervals for the species/blocks.

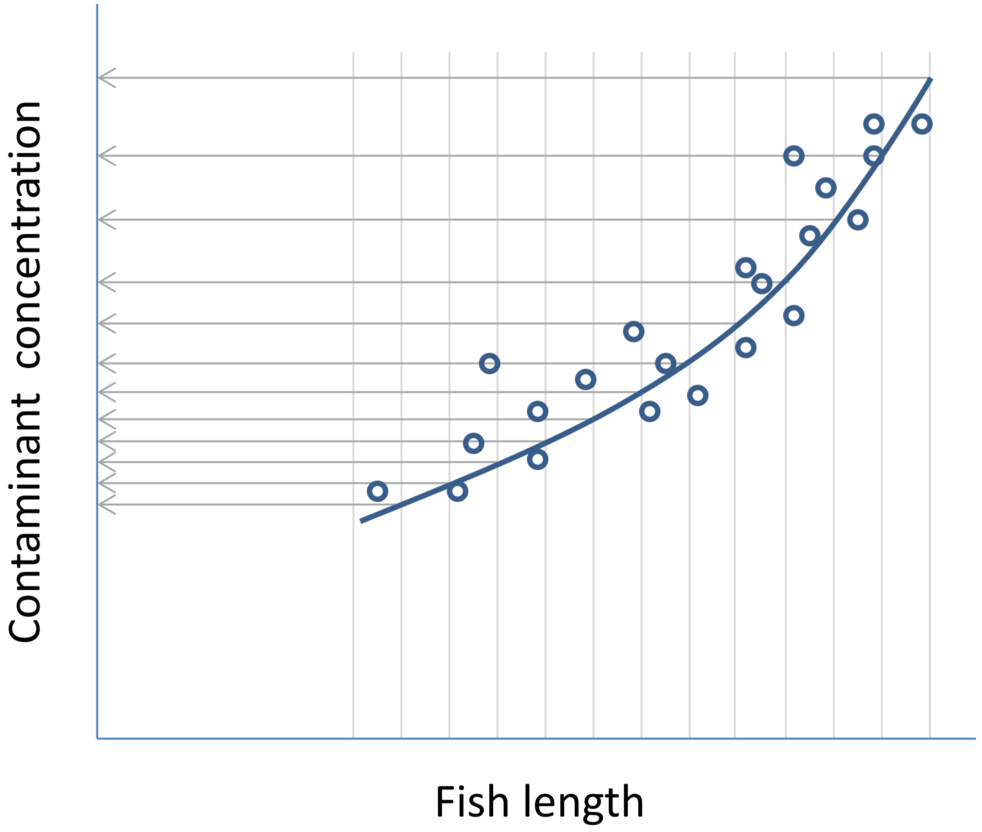

Figure S4: Illustration of a comparison of advisories from the one-chem and multi-chem approaches. The comparison was block-, species- and population- (general and sensitive) specific. The yellow highlighted multi-chem advisories were classified as "more stringent", while the remaining multi-chem advisories were classified as the same.

Simulated fish consumption advisories (meals/month)

Fish Size (cm) →	15	20	25	30	35	40	45	50	55	60	65	70	75	75+
**One-chem**				4	2	2	2	2	2	1	0	0		
**Multi-chem**				2	2	2	2	1	1	1	0	0		

Figure S5: Distribution (in number) of the advisories (meals/month) simulated using the one-chem and multi-chem approaches.

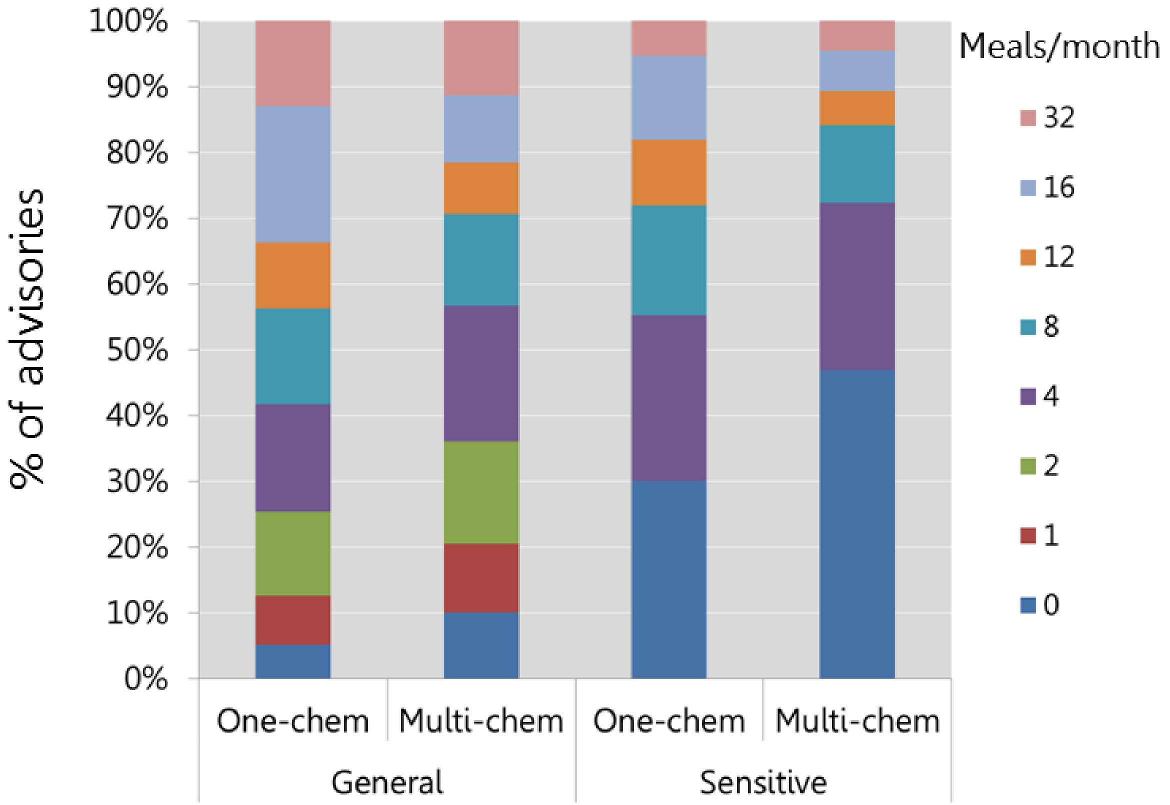

Figure S6: Breakdown (by advisory regions) of percentage of the multi-chem approach based advisories that were more stringent compared to the one-chem approach. Advisory regions are shown in Figure S2. LS: Lake Superior; SMR: St. Mary's River; NC: North Channel (Lake Huron); GB: Georgian Bay (Lake Huron); LE: Lake Erie; LO: Lake Ontario.

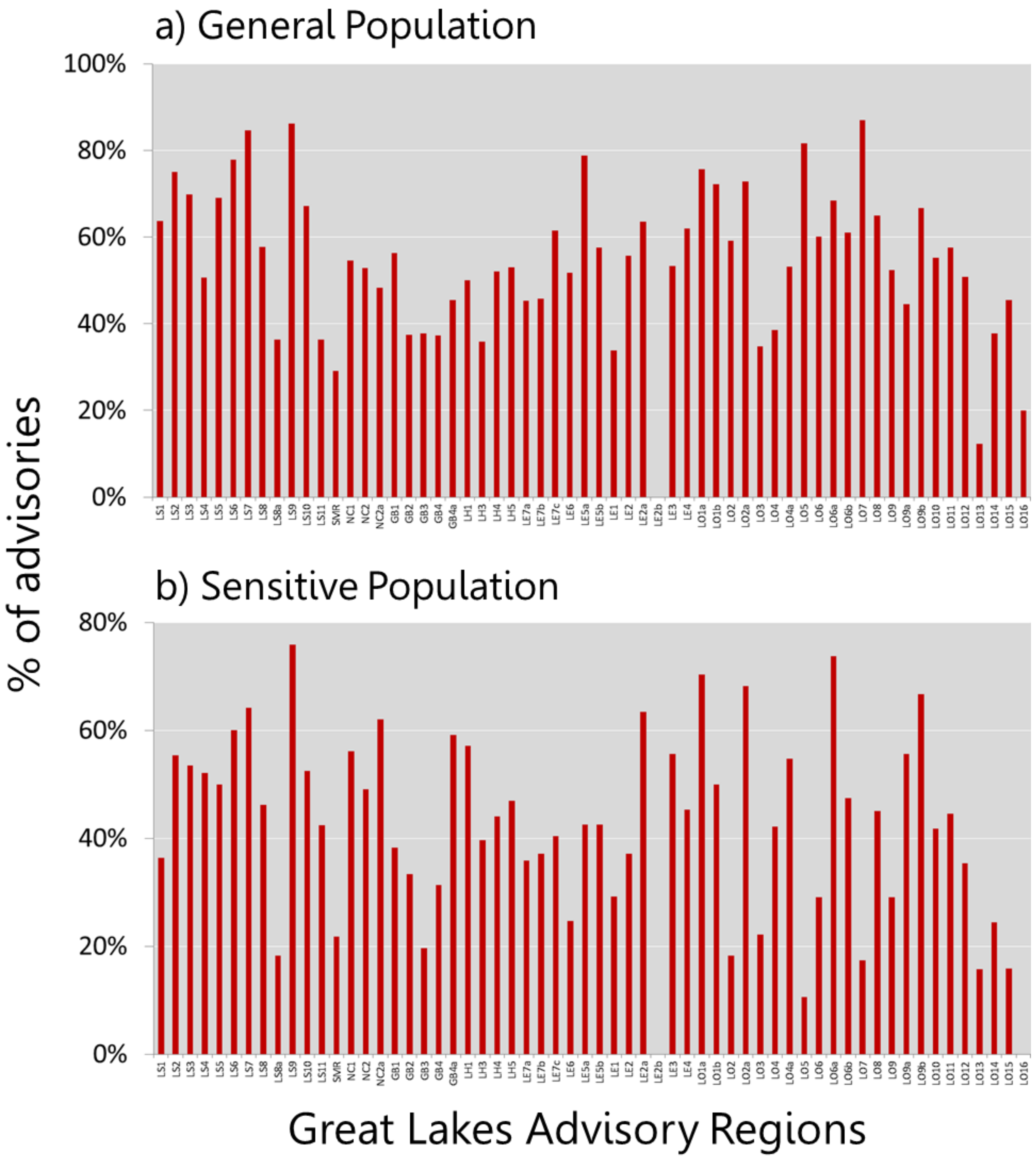

Figure S7: Contaminant-specific Hazard Quotient (HQ) calculated in the multi-chem advisory simulations. The line within the box indicates a median, the box indicates 25th and 75th percentile values, the whiskers indicate the highest and lowest values not classified as statistical outlier values more than 1.5 times away from the interquartile range. Red dotted line indicates an HQ of 1.

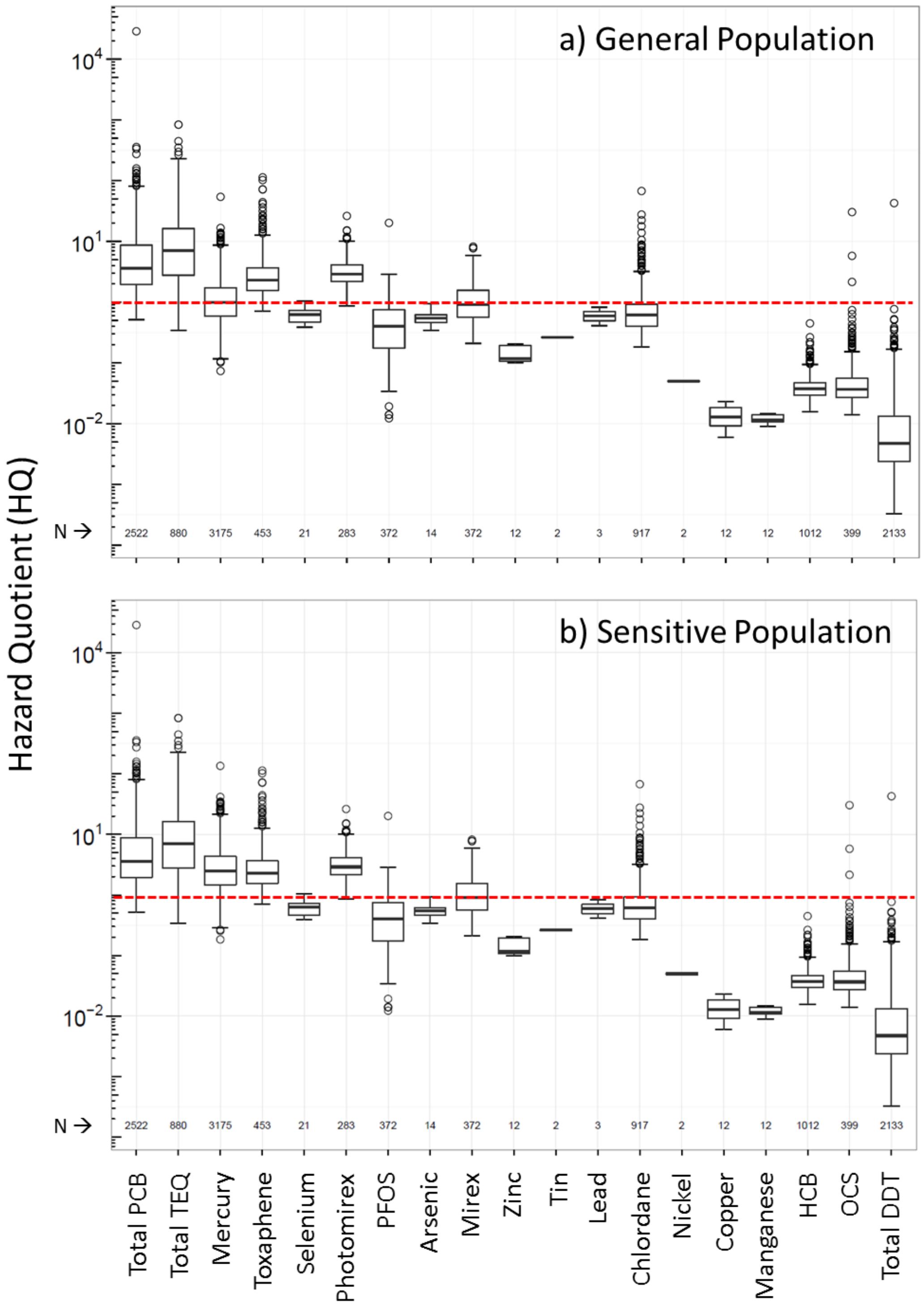

Figure S8:

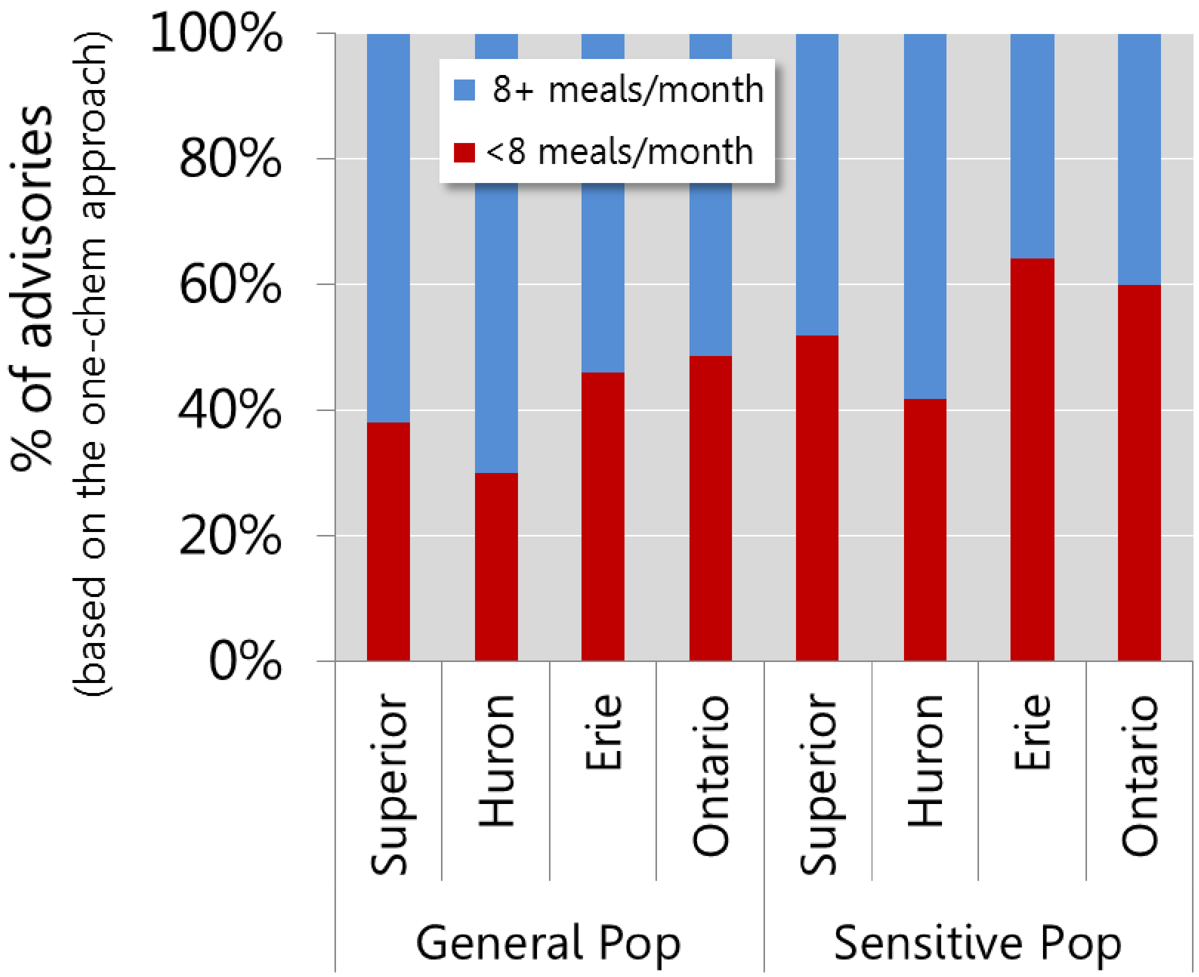
% of advisories
(based on the one-chem approach)
100%
80%
60%
40%
20%
0%
8+ meals/month
<8 meals/month
Superior
Huron
Erie
Ontario
Superior
Huron
Erie
Ontario
General Pop
Sensitive Pop

Figure S9: Percent contribution of contaminant-specific Hazard Quotient (HQ) to the Hazard Index (HI) calculated in the multi-chem advisory approach. Maximum is for the highest contribution of an HQ to HI regardless of a contaminant. The solid circle indicates a mean, the line within the box indicates a median, the box indicates 25th and 75th percentile values, the whiskers indicate the highest and lowest values not classified as statistical outlier values more than 1.5 times away from the interquartile range. Non-detect values were excluded. Similar results for a dataset that included non-detects are presented in Figure 2.

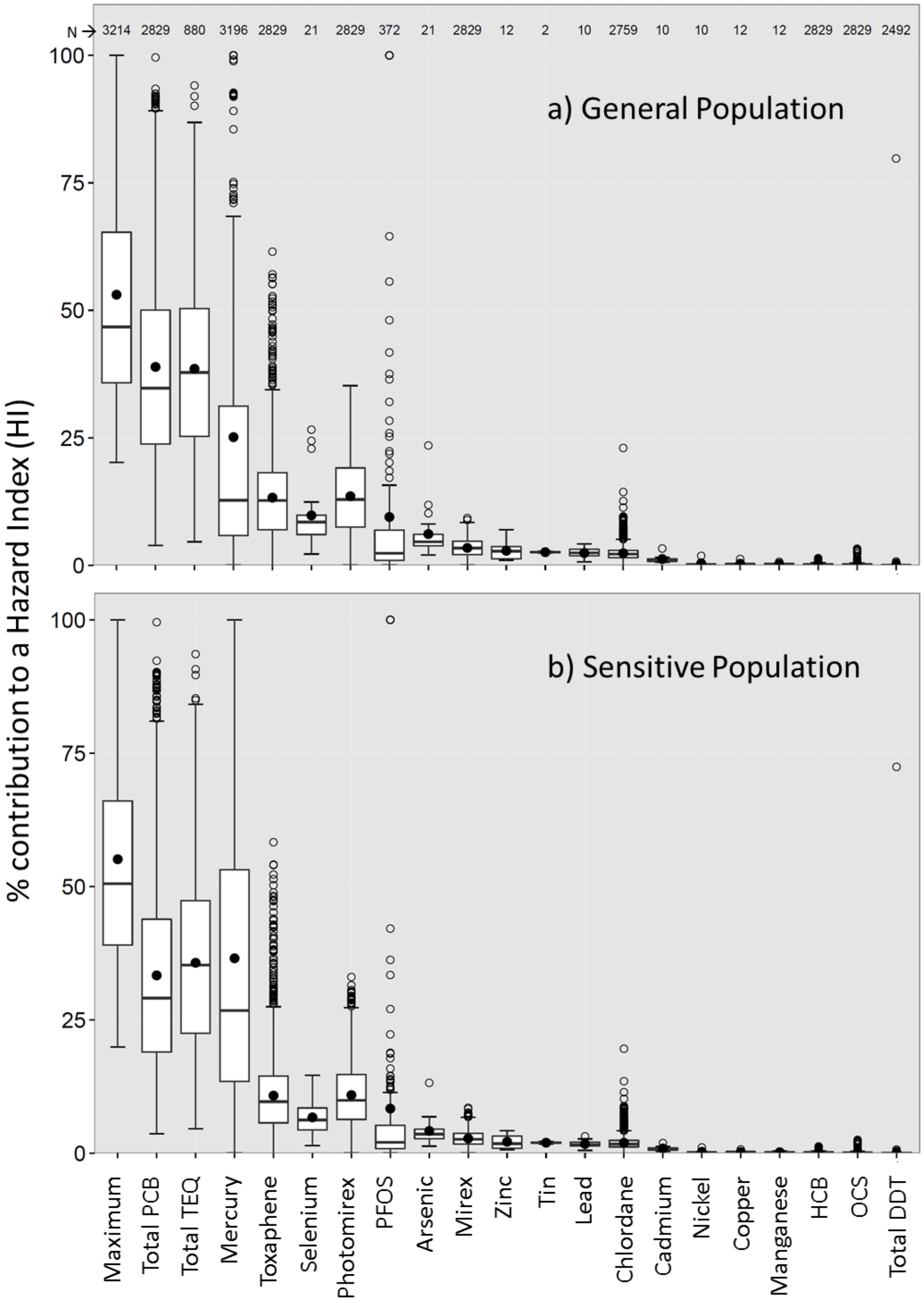

Supplement: (1.5 MB) PDF [file EHP104.s001.acco.pdf]
